# Fossil Worm Burrows Reveal Very Early Terrestrial Animal Activity and Shed Light on Trophic Resources after the End-Cretaceous Mass Extinction

**DOI:** 10.1371/journal.pone.0070920

**Published:** 2013-08-07

**Authors:** Karen Chin, Dean Pearson, A. A. Ekdale

**Affiliations:** 1 Department of Geological Sciences and Museum of Natural History, University of Colorado Boulder, Boulder, Colorado, United States of America; 2 Pioneer Trails Regional Museum, Paleontology Department, Bowman, North Dakota, United States of America; 3 Department of Geology and Geophysics, University of Utah, Salt Lake City, Utah, United States of America; University of Birmingham, United Kingdom

## Abstract

The widespread mass extinctions at the end of the Cretaceous caused world-wide disruption of ecosystems, and faunal responses to the one-two punch of severe environmental perturbation and ecosystem collapse are still unclear. Here we report the discovery of *in situ* terrestrial fossil burrows from just above the impact-defined Cretaceous-Paleogene (K/Pg) boundary in southwestern North Dakota. The crisscrossing networks of horizontal burrows occur at the interface of a lignitic coal and silty sandstone, and reveal intense faunal activity within centimeters of the boundary clay. Estimated rates of sedimentation and coal formation suggest that the burrows were made less than ten thousand years after the end-Cretaceous impact. The burrow characteristics are most consistent with burrows of extant earthworms. Moreover, the burrowing and detritivorous habits of these annelids fit models that predict the trophic and sheltering lifestyles of terrestrial animals that survived the K/Pg extinction event. In turn, such detritus-eaters would have played a critical role in supporting secondary consumers. Thus, some of the carnivorous vertebrates that radiated after the K/Pg extinction may owe their evolutionary success to thriving populations of earthworms.

## Introduction

Life on Earth took a staggering blow at the end of the Cretaceous when innumerable taxa went extinct in both marine and terrestrial environments [1], [2]. Ecosystem responses in the aftermath of the mass extinctions can be examined at well-preserved localities in North America where terrestrial sequences record centimeter-scale changes in physical and biological features across the Cretaceous/Paleogene (K/Pg) boundary [2]. At these sites, latest Cretaceous strata are overlain by a relatively thin (<3 cm) [3], [4] boundary claystone with physical and chemical features indicative of impact ejecta [5]–[8]. The base of this layer is defined as the K/Pg boundary [9]. Analyses of faunal recovery in the earliest Paleocene often focus on vertebrate radiations, but it is difficult to reconstruct ecosystem structure during this interval. Now the discovery of *in situ* burrows very close to the K/Pg boundary in southwestern North Dakota provides new perspectives on the recovery of early Paleocene terrestrial communities.

Previous analyses of multiple terrestrial sequences spanning the Cretaceous/Paleogene boundary have revealed complex patterns of paleoenvironmental changes across the boundary interval. Extensive coal deposits in western North America reflect development of widespread mire environments commencing in the late Maastrichtian and continuing through the early Paleocene [2], [10], [11]. This time-transgressive paleoenvironmental change straddled the impact-defined K/Pg boundary. In the central Great Plains, the increasing wetness may have reflected hydrological changes caused by the transgression of the Cannonball Sea, but the presence of near-boundary coals elsewhere suggests that other factors were involved [2], [11]. Although the facies change pre-dated the end-Cretaceous impact in some areas, analyses of palynological and megafloral assemblages show abrupt plant extinctions across the K/Pg boundary that are independent of local environmental shifts [2], [3], [10]–[14].

Reconstructions of paleobotanical changes during the K/Pg boundary interval provide environmental context for interpreting the paleobiology of the burrows. Despite regional differences in plant communities [10], [13], terrestrial K/Pg boundary localities in North America appear to show similar generalized botanical and habitat responses after the end-Cretaceous impact. Pronounced habitat disruption and widespread extinctions occurred in the immediate aftermath of the impact. This was followed by the development of low diversity mire communities. Spikes in fern spores reflect the dominance of ferns in North America [2], [3], [15], [16] and New Zealand [17], whereas angiosperms were important opportunists at several sites in western Canada [10]. The establishment of more typical Paleocene vegetation took longer and included Maastrichtian mire-adapted taxa that survived the K/Pg extinction [2], [3], [10]–[12].

### Geologic Setting

**Figure 1:**
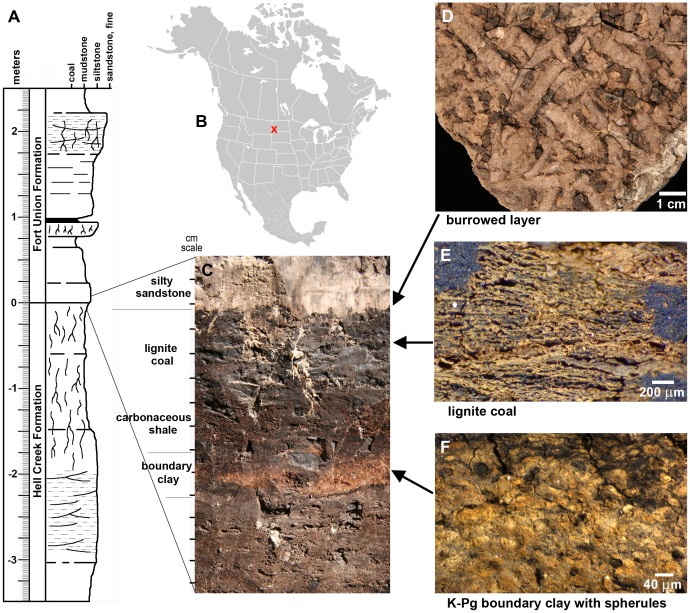
Terrestrial K/Pg boundary site at Mud Buttes, North Dakota. (A) Stratigraphic section through K/Pg boundary interval; this section is located ∼150 m southwest of the Mud Buttes burrow locality (adapted from Bercovici et al. 2009). (B) X marks location of Mud Buttes locality in southwestern North Dakota, USA. (C) View of K/Pg boundary *in situ* at Mud Buttes burrow locality. Note that burrow layer is ∼6 cm above the orange-hued boundary clay at this site. Tic marks at left are in 1 cm increments. (D) Plan view of the undersides of burrows at lignite/silty sandstone interface (specimen KT4/UCM 98213). (E) Close-up of lignitic coal showing poorly-compacted plant debris. (F) Close-up of boundary clay showing abundant spherules ∼40 µm in diameter.

**Figure 2:**
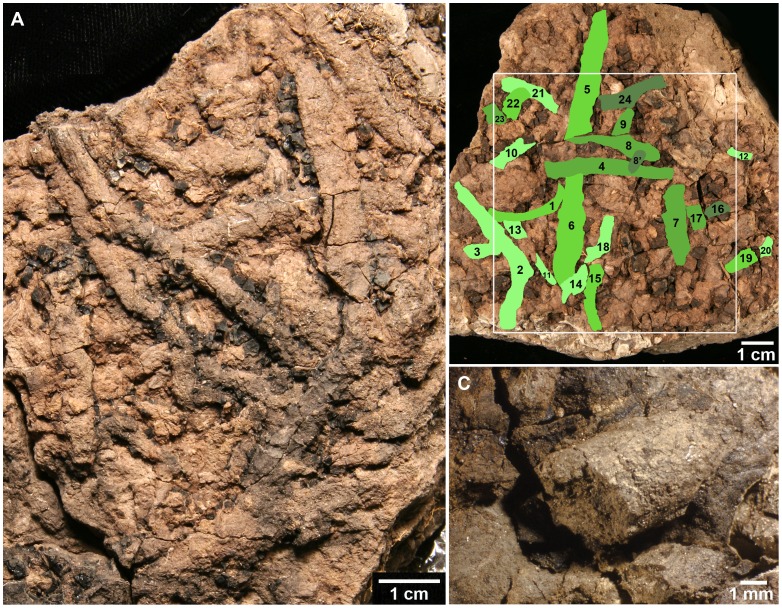
*Planolites* isp. burrows from Mud Buttes. (A) Plan view showing undersides of closely packed horizontal burrows at coal/silty sandstone interface. Note coal still adherent to some burrows (specimen KT3/UCM 98212). (B) Discernible burrows colored and numbered to illustrate minimum burrow density within 8 cm^2^ (specimen KT4/UCM 98213). Portions of at least 23 burrows comprise roughly 30% of area of white box. Burrows are colored different shades of green to illustrate overlapping relationships. The burrowing activity likely took place within a short period of time, but burrows colored darker green were lower in the soil profile and appear to have been crossed *in situ* by other burrows (lighter shades of green). Burrows colored lightest green were the topmost burrows. Note that this specimen is the same as that in Figure 1D. (C) Close-up of burrow 19 in B (specimen KT4/UCM 98213).

The Mud Buttes Cretaceous-Paleogene boundary site is located at the southern end of a series of K/Pg boundary intervals exposed along an ∼50 km transect parallel to the Little Missouri River in southwestern North Dakota [13] (Figure 1A,B). The boundary layer at this site has been identified as an impactite by the presence of shocked quartz [18], an iridium anomaly [13], and spherules [2] (Figure 2C,F). Moreover, the boundary claystone at Mud Buttes is coincident with the facies change at the contact between the latest Maastrichtian Hell Creek Formation and earliest Paleocene Fort Union Formation (Ludlow Member) [11].

An initial microstratigraphic and palynological analysis of the boundary sequence at Mud Buttes [13] revealed that the boundary claystone is overlain by a thin (∼1 cm) lignite, followed by 8 to 9 cm of carbonaceous shale and mudstones. This study documented high percentages of fern spores above the boundary claystone: 56.5% in the thin lignite, 88.0% in the lower carbonaceous shale, 67.0% in the upper carbonaceous shale, and 38.0% in the overlying mudstone (1, 2, 10, and 14 cm above the boundary clay respectively). Local topographic or environmental variation, however, is demonstrated by a second investigation of an adjacent boundary sequence in the Mud Buttes area [14], which lacked both a boundary-associated lignite and fern spike. Nevertheless, palynological analysis of a siltstone approximately 12 cm above the boundary showed a moderate spike in percentages of freshwater algae, quillwort, and bryophyte spores. Including fern spores (∼40%), this palynological assemblage is dominated (∼75%) by aquatic or wet environment-associated plants.

## Methods

Several poorly consolidated, burrow-bearing blocks were collected from just above the K/Pg boundary at the original Mud Buttes locality on U.S. Bureau of Land Management land. No permits were required for the described study, which complied with all relevant regulations. The diameters of 50 burrows from four blocks were measured, and the burrow density of an 8 cm square on one well-burrowed slab was assessed with ImageJ software. A few pieces of the burrow layer were embedded with acetone-thinned epoxy so thin sections could be made; photomicrographs were taken with Leica DMR and MZ 12.5 microscopes and a SPOT RT digital camera. Two thin sections were polished, carbon-coated and examined with the QEMSCAN (Quantitative Evaluation of Minerals by SCANning electron microscopy) automated mineralogy system at the Colorado School of Mines. These samples were analyzed with a 5 nA beam current for mineral modal abundance (at 25 kV) and organic carbon (15 kV) with beam stepping intervals of 20 µm (bioturbated sediment specimen KT3-T2b/UCM 98212) and 10 µm (burrow fill specimen KT3-T3b/UCM 98212). Burrow-bearing slabs and associated thin sections are reposited in the University of Colorado Museum of Natural History paleontology collections.

A block of the poorly consolidated sediments of the boundary section was also examined. The approximately 30×30×35 cm block had been collected in a plaster jacket from the same site, and was opened with a wood saw to examine the intact microstratigraphy.

## Results

The microstratigraphy of the K/Pg boundary interval examined in this study shows small-scale differences from the section first described at Mud Buttes [13], even though the two sites were less than three meters from each other. The sediments of this burrow-bearing interval (hereafter referred to as the “burrow locality”) transition upward from the boundary claystone to carbonaceous shale, lignite, and a light-colored silty sandstone (Figure 1C,D); no disconformities are evident. At the *in situ* boundary exposure at the burrow locality, the distance from the top of the boundary clay to the burrow layer was measured at ∼6 cm (Figure 1C). The nearby boundary section recovered in the jacketed block was comparable, with ∼5.4 cm separating the boundary clay from the burrow layer (0.6−1 cm of carbonaceous shale plus 2.3−4.4 cm of lignite).

The fossil burrows are preserved as three-dimensional casts at the contact between the lignite and the overlying silty sandstone (Figure 2). The straight to sinuous burrows are conspicuous at the facies change, but are poorly consolidated and break apart easily. All are horizontal and have no preferred orientations. The burrows are somewhat compressed, but appear to exhibit fairly consistent widths with no noticeable tapering; diameters range from 2.0−10.1 mm, with a mean of 4.8 mm (n = 50). No meniscate backfill structure is evident. Most examined burrows do not branch, but three forked burrows were observed.

**Figure 3:**
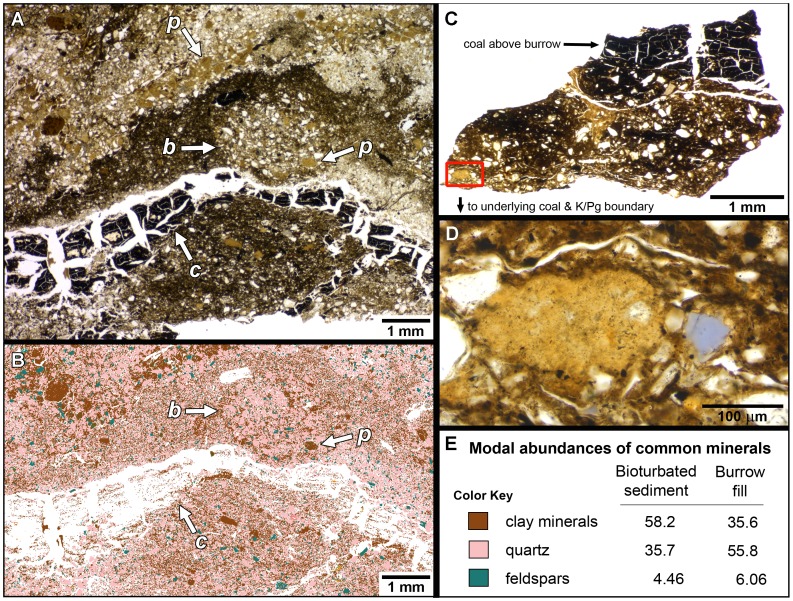
Thin sections of bioturbated sediment (A, B) and a section of burrow fill (C, D). (A) Photomicrograph of thin section of epoxy-impregnated bioturbated layer (specimen KT3-T2b/UCM 98212). Bottom of photo is at the coal/silty sandstone interface, and top is several mm higher in section. The ∼3 mm diameter circular structure labeled “*b*” in the center of the photograph appears to be a cross section of a burrow. A thin layer of fractured coal is evident below this (“*c*”). Numerous pelletoid blebs are visible, including one in the burrow cross-section, and several others massed together in the upper part of the photo (two are labeled “*p*”). (B) QEMSCAN (Quantitative Evaluation of Minerals by SCANning electron microscopy) image of portion of thin section in A showing distribution of clay minerals (brown), quartz (pink), and feldspars (turquoise) in the bioturbated sediment (note that organic matter was not detected at the kV setting used to analyze minerals). Concentrated blebs of clay are conspicuous. Same letter designations apply as in A. (C) Thin section of partial burrow fill from KT3-T3b/UCM 98212. This burrow was sandwiched between the underlying coal and a thin layer of coal on top, indicating burrowing within the Paleocene peat. Red box in lower left shows site of photomicrograph in D. (D) Close-up of area of red-box in C showing a pelletoid clay mass containing tiny (<25 µm) mineral clasts; this clay bleb appears to be a fecal pellet from a deposit-feeding invertebrate. (E) Color key of minerals in QEMSCAN image B and calculated QEMSCAN mass percentages of minerals in bioturbated sediment sample (portion of which shown in B, and in burrow fill shown in C.

Plan views of the contact between the lignite and silty sandstone show multiple closely-packed burrows. Burrows commonly overlap (Figure 2B); at one junction, three stacked burrows are evident. Much of the sediment between the burrow casts appears to have a similar texture as the burrow fill (Figure 3). This suggests that bioturbation and overprinting of older burrows likely obscured overall burrow density. Image analysis indicates that the density of recognizable burrows in one 8 cm^2^ sample is ∼30% (Figure 2B). Although burrows are evident at the facies contact with the underlying coal, some burrows also have a thin layer of coal on their upper surfaces, indicating burrowing within the precursor peat (Figure 3C).

Clastic sediment grains are embedded in the rough outer surfaces of the burrow casts. Light-colored impressions of plant fragments are also haphazardly distributed on the surfaces of some burrow casts, but the orientation of the plant tissues rarely corresponds to the direction of the burrows; some plant impressions extend from one burrow cast to another.

Thin sections of the burrowed layer show a heterogeneous silty sandstone with abundant organic matter (Figure 3A). QEMSCAN analysis of the bioturbated sediment reveals a predominance of angular quartz grains and dispersed clay minerals, with scattered feldspathic clasts (Figure 3B). This section includes what appear to be burrow cross-sections with more quartz clasts than the host sediment. Quantitative comparisons of minerals in the bioturbated host sediment and those in a nearby burrow fill (∼8 cm away) show that the burrow fill contains more quartz (mass percentage ∼56% versus 36%), and less illite (muscovite and smectite together comprise ∼22% versus 46%; Figure 3E). QEMSCAN analysis also indicates high percentages of organic matter in both samples: ∼12.9% in the burrow fill and ∼18.5% in the bioturbated sediment (∼7.7% measured in an area of host sediment that lacks patches of coal).

Microscopic analysis of the bioturbated layer also shows numerous pelletoid masses that are commonly ∼200 µm on a side. Petrographic and QEMSCAN analysis reveal that these are aggregates of concentrated clay minerals containing tiny (∼25 µm diameter) feldspathic or siliceous clasts (Figure 3B,D). The blebs are patchily distributed, and are found in both burrow fills and bioturbated sediment.

## Discussion

### Stratigraphic and Paleoenvironmental Context

The undisturbed boundary sequence and *in situ* burrows at the Mud Buttes locality demonstrate animal activity within six vertical cm of the K/Pg impactite. Both sedimentary context and palynology of the site indicate that the burrowers inhabited a wetland environment. The thin layer of coal directly below the burrows reflects mire conditions in which plant growth exceeded decomposition; this is consistent with the abundance of fern spores documented in the earliest Paleocene sediments at the original Mud Buttes locality [13]. The facies change from lignite to silty sandstone indicates inundation of the coal-precursor wetland with clastic sediment. Higher percentages of quartz grains in the analyzed burrow fills relative to the bioturbated sediment may reflect sedimentological and/or biotic processes. Open burrows might have been passively filled during clastic input, or deposit feeding behavior may have modified burrow contents. Nevertheless, the preponderance of aquatic pteridophyte spores recovered from ∼12 cm above the boundary clay at the second Mud Buttes site [14] indicates that the local environment remained wet when the burrows were created.

The amount of time elapsed between the end-Cretaceous impact and construction of the Mud Buttes burrows can be gauged by considering sediment accumulation rates. Clastic sedimentation rates in the Early Paleocene have been estimated by correlating the estimated duration of the Paleocene portion of subchron C29r with the thickness of Paleocene sediments accumulated during this subchron in the Rocky Mountains. Sediments in a core drilled in the Denver Basin [19] were used to estimate that the sediment accumulation rate during the first 270,000 years of the Paleocene in Colorado was approximately 129.6 m per million years [20], or 1 cm per 77 years. Another study assembled a composite section of Paleocene sediments from nine K/Pg boundary sites in southwestern North Dakota (all within 60 km of Mud Buttes) and calculated a sediment accumulation rate of 79–89 m per million years for the Ludlow Member of the Fort Union Formation (the first 2.31 to 2.61 million years of the Paleocene) [21], or 1 cm per 112 to 127 years.

Accumulation of the coal layer would have taken longer. McIver [16] used estimated rates of peat accumulation in the Okefenokee Swamp [22] and approximations of peat-to-lignite coal compaction ratios (2.5∶1 to 10∶1) [23], to estimate that it took roughly 80 to 320 years to accumulate 1 cm of coal from aquatic herbaceous plant material above the K/Pg boundary in Saskatchewan.

These Early Paleocene sediment and coal accumulation rates suggest that burrowing at the Mud Buttes locality took place within a relatively short period of time after the end-Cretaceous bolide impact. Using the slower estimated rates of clastic accumulation in the early Paleocene of North Dakota, the approximately 1 cm of carbonaceous shale plus the 5 cm of lignitic coal between the burrows and spherule-rich K/Pg may have accumulated in 512–1,727 years. Although the horizontal burrowing occurred below the ancient soil surface, it seems unlikely that these burrows were very deep, because the burrow patterns suggests terrestrial deposit feeding (see discussion below) which is usually done in upper soil layers that contain appreciable organic matter. If we presume that the burrows were 1 to 20 cm below the sediment surface, we must add an additional 127 to 2,540 years to the time represented by the six cm between the boundary clay and the burrows. Thus, we can estimate that the burrowing activity at the Mud Buttes locality took place within 650 to 4,300 years of the end-Cretaceous impact. These rates are approximations, but even if the upper figure in this range were doubled, the documentation of significant animal activity within ten thousand years of a catastrophic extinction event is remarkable.

### Likely Fecal Pellets in the Burrowed Layer

The size, shape, and distribution of the small pelletoid clay blebs in the bioturbated burrow horizon suggest that these structures are fecal pellets. Their clay composition with silt-sized mineral inclusions is consistent with feces produced by extant organisms that ingest large quantities of sediment. Studies of feces produced by deposit-feeding lugworms [24], [25] and earthworms [25] have demonstrated that sediment ingestion increases rates of both mineral weathering and production of authigenic clays.

### Identity of the Burrowers

The fossil burrows at the Mud Buttes locality can be classified as *Planolites* isp., based on their simple, unlined cylindrical structure, horizontal meandering habit, and absence of systematic branching [26]. This ichnotaxonomic designation does not indicate which type of animal did the burrowing, but the size, structure and patterns of the burrows suggest that they were created by worms that actively fed as they moved through the substrate. The density and overlapping nature of the burrows suggest ample food resources and essentially contemporaneous activity.

The occurrence of the burrows at the interface of the coal and overlying, silty sandstone, the fact that some burrows are completely emplaced in coal, and the abundance of wet environment-associated palynomorphs suggest that the burrowers were able to tolerate the adverse conditions often associated with wetlands, such as low oxygen concentrations, repeated inundation [27 28], low pH, and biotic and abiotic toxins [27]. Nevertheless, it is likely that the influx of clastic sediment mitigated some wetland stressors, allowing the burrowers to colonize the changing habitat.

In modern terrestrial environments, earthworms (clitellate annelids) produce burrows that are comparable to the fossil burrows at Mud Buttes in terms of size, structure, and depositional context. Active earthworm burrows are open, cylindrical structures [29], [30], and burrow systems can vary in depth or extent of branching with different taxa or soil conditions [31], [32]. Earthworms generally deposit their fecal pellets in or near their burrows [29]. Furthermore, earthworms can inhabit wetland environments [29], [33]; some taxa have been observed to withstand over 100 days in inundated soils [34]. Thus several lines of evidence strongly suggest that earthworms created the Mud Buttes burrows.

The fossil record of soft-bodied worms is scant, but it appears that earthworms were present by the Cretaceous, if not before. Body fossils of possible oligochaetes have been found in Permian, Jurassic, and Cretaceous deposits [35]. Particularly relevant is a cast of the tapered end of a likely earthworm recovered in Wyoming from the base of the Fort Union Formation [36], the same formation that hosts the Mud Buttes burrows; this fossil clearly shows ring-like sections that resemble the annuli of extant earthworms. The morphological evidence for the presence of Cretaceous earthworms is supported by molecular clock data that suggest that Mediterranean hormogastrid earthworms diverged in the Late Cretaceous, around 97 to 67 million years ago [37].

Trace fossils attributed to ancient earthworms have been described from Triassic [38], Jurassic to Early Cretaceous [39], Eocene [40], [41], and Pleistocene [42] sediments. Fossil fecal pellets were observed in the Triassic burrows [38], and reported Eocene examples are packed with so many fecal pellets that the burrows have conspicuously bumpy exteriors and have been assigned to the ichnogenus *Edaphichnium* [40], [41].

Although we are unable to identify the genus or species of the Paleocene Mud Buttes burrowers, we can infer the ecological niche of the burrowers based on burrow structure. Earthworms are commonly categorized by ecological groups that reflect feeding and burrowing habits. Epigeic earthworms generally feed on plant litter and rarely burrow. Anecic earthworms feed on plant litter at the surface or in the topsoil, but commonly construct deep permanent burrows; they tend to deposit fecal castings at the soil surface. Endogeic earthworms tend to be geophagous, and deposit castings in and around their burrows as they move through the sediment in extensive horizontal burrows that are rarely re-used [29], [43], [44]. Endogeic burrows are also less durable than continually used anecic burrows [45]. Thus the poorly consolidated and horizontal Mud Buttes burrows associated with sporadically distributed fecal pellets were likely produced by endogeic earthworms.

### Paleoecological Implications

The fragile fossil burrow networks at Mud Buttes could not have been transported, and the horizontal orientation of the burrows demonstrates infaunal animal activity at the stratigraphic level at which they are found. This not only reveals some of the earliest-known terrestrial animal activity after the end-Cretaceous impact, but also provides rare evidence for the presence of soft-bodied animals in the earliest Paleocene. The closely-packed and overlapping burrows reflect robust activity in the soil by a flourishing invertebrate population.

Survivors of the end Cretaceous extinction event were able to withstand both short- and longer-term repercussions of the bolide impact and ongoing Deccan vulcanism. Global thermal radiation caused by re-entry of asteroid impact ejecta apparently presented an immediate and potentially deadly, short-term consequence of the impact [8]. Robertson et al. [46] suggest that this intense pulse of infrared radiation would have killed exposed organisms, and infer that survivors were protected within burrows, natural cavities, or bodies of water. Earthworms not only live in the soil, but are also capable of employing several mechanisms that allow them to withstand unfavorable environmental conditions: their protective egg-bearing cocoons can remain unhatched until conditions improve, and the worms can enter a state of quiescence or diapause, or can burrow deeply to avoid adverse conditions near the sediment surface [43].

Large impact- [8] and volcanism-caused [47] releases of particulates and gases are thought to have triggered other major environmental perturbations such as pronounced acid rain and reduced sunlight. Some of these effects probably served to reduce photosynthesis [8], thereby adversely impacting secondary consumers that depend on food webs based upon active primary producers. Sheehan et al. [48] suggested that detritivores would have thrived on existing stores of dead and decomposing carbon resources despite decreases in primary production.

Given the predicted consequences of the K/Pg bolide impact, detritivorous/geophagous earthworms burrowing in organic-rich sediments fit the criteria of likely Paleocene survivors. In turn, these burrowers would have served as prey for other animals that survived the K/Pg mass extinction; many birds, non-avian reptiles, amphibians, mammals, fish, and invertebrates eat earthworms in modern ecosystems [29], [34], [43]. Analyses of fossil leaves from Upper Cretaceous to Paleocene sediments reveal a decrease in specialized insect damage, suggesting substantive extinctions of herbivorous insects at the end of the Cretaceous [49], [50], and a diminution of their roles in early Paleocene ecosystems. With a reduction in phytophagous insect prey, survival of the Mud Buttes burrowers would have been particularly important in supporting small secondary consumers in the early Paleocene.

### Conclusions

Fossil burrows have provided information on the rapid recovery of marine ecosystems after the K/Pg extinction event at sites in Europe [51], [52], [53]. But the Mud Buttes fossil burrows offer rare perspectives on the recovery of soft-bodied terrestrial fauna from a North American site that was much closer to the Chicxulub impact site (∼ 2,700 km versus 8,200 to 10,200 km for the European sites) [8]. Although the nature and severity of the physical effects of the end-Cretaceous impact continue to be debated [8], the survival of subterranean, detritivorous burrowers is consistent with the postulated consequences of both the extraterrestrial impact and prolonged vulcanism. Moreover, these organisms were able to exploit the widespread mire conditions of the early Paleocene of North America. Much attention has been focused on the radiation of terrestrial vertebrates in the early Paleocene, but the survival of carnivorous mammals, birds, and other vertebrates in the early Cenozoic would have depended on the availability of prey that were not decimated by interruptions in primary productivity. The Mud Buttes burrows suggest that robust populations of annelids likely provided key detritivore-based sources of protein for survivors of the end-Cretaceous catastrophe.
